# Clinical and Laboratory Diagnosis of Antiphospholipid Syndrome: A Review

**DOI:** 10.7759/cureus.61713

**Published:** 2024-06-05

**Authors:** Muhamad Aidil Zahidin, Salfarina Iberahim, Mohd Nazri Hassan, Zefarina Zulkafli, Noor Haslina Mohd Noor

**Affiliations:** 1 Department of Hematology, School of Medical Sciences, Universiti Sains Malaysia, Kota Bharu, MYS

**Keywords:** human pathophysiology, anti-β2-glycoprotein i antibodies, lupus anticoagulant, anti-cardiolipin antibodies, antiphospholipid antibody syndrome (aps)

## Abstract

The antiphospholipid syndrome (APS) manifests through venous or arterial thrombosis, with or without pregnancy complication alongside the continuous presence of antiphospholipid antibodies (aPL). APS classification relies on three aPL subtypes: anticardiolipin (aCL), anti-β2-glycoprotein I antibodies (anti-β2GPI), and lupus anticoagulants (LA) antibodies. Given that thrombosis and pregnancy issues are not unique to APS, the precise and reliable identification of aPL forms the basis for diagnosis. Semi-quantitative solid-phase assays identify two antibodies, aCL and anti-β2GPI, while LA detection occurs through various phospholipid-dependent coagulation assays that are based on antibody behaviour. LA, specifically, is conclusively associated with thrombosis, prompting discussions around the serological criteria for APS. Despite advancements in LA detection, the standardisation of all aPL detection assays remains imperative. The combined presence of aCL and anti-β2GPI with thrombosis inconsistently triggers concern. Initial presentations by APS patients commonly exhibit a heightened risk of stroke, miscarriages in the later stages of pregnancy, positive results of LA tests, and widespread thrombosis across multiple organs, often leading to adverse outcomes. Correctly diagnosing this condition is pivotal to avoid unnecessary long-term secondary thromboprophylaxis.

## Introduction and background

Antiphospholipid syndrome (APS) is a condition characterised by an autoimmune response that leads to prothrombotic disorder. This syndrome is closely linked to the presence of antiphospholipid antibodies (aPL), which are immune proteins that mistakenly target certain components of cell membranes. Initially identified and described by physician Graham R. V. Hughes in 1993, the syndrome is also commonly referred to as Hughes’ syndrome in recognition of his seminal contribution to its understanding [1]. APS is identified by venous, arterial, or small vessel thrombosis. Additionally, it encompasses recurrent early pregnancy loss, fetal loss, or pregnancy complications in the presence of persistent documented aPL, such as lupus anticoagulants (LA), anti-β2-glycoprotein I antibodies (anti-β2GPI), or moderate-to-high titer anticardiolipin (aCL) antibodies. APS patients often exhibit a heightened risk of stroke, miscarriages in the later stages of pregnancy, positive results in LA tests, and widespread thrombosis across multiple organs, leading to unfavourable outcomes. Correctly diagnosing this condition is paramount to prevent unnecessary long-term secondary thromboprophylaxis.

## Review

Detection of APS

Early detection of APS is crucial to prevent recurrent thrombosis because patients face a significant risk of recurrence, with rates of 52-69% over five to six years, especially within the first few months after discontinuing anticoagulation [2]. Managing recurrent thrombotic events can be challenging, often requiring extended or lifelong antithrombotic therapy. Up to 20% of deep vein thrombosis (DVT) cases, with or without pulmonary embolism, may be linked to aPL, highlighting the importance of prompt anticoagulation therapy upon early detection [3]. Confirming APS requires two positive aPL tests at least six weeks apart to ensure persistent aPL and accurately assess thrombotic risk. While asymptomatic individuals with transiently elevated aPL levels have a low risk of thrombosis, those with persistently positive aPL are at higher risk and may benefit from prophylactic therapy [2,4].

Prevalence of APS

In the USA, APS has an annual incidence of 2.1 per 100,000 and a prevalence of 50 per 100,000 adults, predominantly affecting the White population [5]. In Argentina, the incidence is 2.6 per 100,000, with a prevalence of 40.5 per 100,000 [5]. South Korea reports a lower incidence of 0.75 per 100,000 and a prevalence of 6.19 per 100,000 [5]. In Taiwan, the incidence rose from 4.87 to 6.49 per 10,000 person-years between 2000 and 2013, with women showing a higher incidence than men [6]. Overall, the prevalence of APS in the general population is about one in 2,000, making it a rare disease with an incidence of approximately five per 100,000 person-years and a prevalence of 40 to 50 per 100,000 person-years [7]. This rarity underscores the need for further research and awareness.

In healthy individuals, aPL may occur incidentally, with less than 10% having low-titre aCL and less than 1% exhibiting moderate-to-high levels of titre aCL and/or positive LA test. The prevalence of positive aPL tests tends to increase with age. Diagnosing APS in older patients requires particular care due to the broader differential diagnosis of vascular occlusion compared to young adults [8]. Roughly, 10% of healthy blood donors exhibit positivity for aCL antibodies, and approximately 1% test positive for LA. However, less than 1% of these samples remain in a positive status after one year [9].

Around 30% to 40% of individuals diagnosed with systemic lupus erythematosus (SLE) and roughly 20% of rheumatoid arthritis (RA) patients test positive for aPL [8]. Among patients with SLE, about 20% to 30% exhibit recurrent aPL profiles, which are linked with a heightened risk of clinical complications [9]. Among individuals without autoimmune disease, the prevalence of aPL positivity is approximately 17% in stroke patients younger than 50 years old, 11% in patients who had myocardial infarction, 10% in patients with venous thrombosis, and 6% in women experiencing pregnancy complications. However, it is important to note that these prevalence estimates primarily stem from studies that involved aPL testing conducted once, included borderline positive results, or both. Robust studies employing stringent criteria for aPL and clear definitions of clinical events are necessary [9].

Clinical features of APS

The clinical characteristics of APS vary widely, ranging from mild conditions like asymptomatic aPL positivity to severe cases such as catastrophic APS. Venous and arterial thrombosis, along with pregnancy-related complications, are considered the defining features of the disease [10]. The presence of aPL can manifest in various clinical scenarios, ranging from asymptomatic individuals identified as “aPL carriers” to those with classic APS. Classic APS is characterised by recurrent vascular events such as arterial or venous thrombosis. Additionally, APS can predominantly affect pregnancy, leading to obstetric APS. Furthermore, there exist patients who test positive for aPL but display clinical manifestations that are not thrombotic or obstetric in nature. A small subset of APS patients may experience catastrophic APS, an aggressive and life-threatening form marked by the rapid onset of multiple occlusive events that cause multiorgan failure [1].

The classification of ‘primary’ APS is when it occurs without any association with other underlying diseases, whether it is termed “secondary” when linked to conditions, especially autoimmune disorders like SLE. Although aPL positivity has been observed in connection with malignancies, infections, and medications, in such instances, aPL titers typically remain transient and low. Consequently, they do not significantly elevate the risk of adverse pregnancy or thrombosis outcomes. While the clinical forms of primary and secondary APS can bear similarities, secondary features may be further complicated by the manifestation of the underlying disease [1].

Beyond thrombosis and pregnancy complications, several other clinical conditions have been tentatively associated with aPL. These include heart valve disease (often occult), thrombocytopenia, livedo reticularis/racemose, chorea, and nephropathy. However, similar to the thrombotic and pregnancy-related symptoms, none of these conditions are specific to APS [11]. Transverse myelopathy, a condition seen in SLE, might occur more frequently in individuals with aPL [12]. There have been claims regarding an association between APS and infertility, although such assertions lack substantial evidence [13]. The relationship between APS and migraines remains controversial, with some studies indicating a connection [14], while others have found no such association [15,16]. Another contentious notion is the resemblance of APS to multiple sclerosis, as suggested by Hughes in 2003 that APS might respond to anticoagulant therapy [17]. However, while aPL may be detected in certain instances of otherwise typical multiple sclerosis [18], it could represent an epiphenomenon in a disorder primarily driven by immune mechanisms.

Additionally, the term ‘seronegative APS’ was introduced to characterise patients exhibiting clinical manifestations of APS yet consistently test negative for conventional aPL [19]. In such instances, clinicians should consider the possibility of ‘seronegative APS’ to ensure appropriate therapy and thereby improve prognosis [20]. However, diagnosing 'seronegative APS' could pose challenges as the primary manifestations of APS, such as pregnancy failure and thrombosis, are usual and often lack an autoimmune basis, could be challenging [21].

Pathophysiology of APS

Although not all individuals with aPL develop APS, a robust correlation exists between aPL presence and conditions like venous thrombosis, ischemic stroke, and myocardial infarction. The likelihood of developing clinical APS may hinge on various factors such as antibody profile - comprising type, titer, and underlying comorbidities. For instance, individuals demonstrating triple positivity with positive LA, along with high anti-B2GPI antibodies and titers of aCL face a heightened risk of APS onset. Conversely, sporadic positivity or isolated or low titers of aCL and anti-B2GPI antibodies pose a relatively lower risk. Patients with SLE, concurrent cardiovascular risk factors, a history of recurring thrombotic events despite undergoing anticoagulation therapy, or an arterial thrombosis background are at an elevated risk for experiencing recurrent thrombosis [10].

In experimental animal models, aPL from individuals with the syndrome has been demonstrated to directly contribute to the development of thrombotic manifestations [22]. A few hypotheses have been put forth to elucidate the molecular foundation of the prothrombotic state linked to state antibodies [22,23]. Reports indicate that aPL binds to and triggers activation of endothelial cells, impedes natural anticoagulant pathways, disrupts annexin V binding to anionic phospholipids, and interferes with fibrinolysis [22,23]. However, the precise clinical significance of individual pathways remains unclear.

Recognised as pathogenic entities, aPL play a crucial role in thrombosis rather than merely serving as serological marker of aPLs. The 'two-hit' theory suggests that the development of APS involves a two-step process. The first step (initial hit) is characterised by the activating of a prothrombotic or inflammatory response triggered by aPL. This initial hit sets the stage for heightened vulnerability to thrombotic events. The second step (second hit) occurs when the individual is exposed to an immediate precipitating event, which could include undergoing surgery, being exposed to exogenous oestrogen, experiencing immobilization, or going through pregnancy. Intriguingly, in the context of pregnancy, it is not solely a precipitating prothrombotic state; examination of conception products from aPL-positive and aPL-negative women with recurrent early miscarriage highlights a clear deficiency in the invasion of decidual endovascular trophoblasts in obstetric APS (OAPS) and indicates that placental infarction is not exclusively to APS [24].

Increasing experimental evidence suggests a non-thrombotic role in the pathogenesis of OAPS through aPL-mediated activation of the complement, inflammation, and disruption of placental development and function. However, clinical data from the European Registry of Obstetric Antiphospholipid Syndrome (EUROAPS) involving 247 OAPS patients indicate a lower likelihood of progressing to SLE and thrombosis in comparison to thrombotic APS patients, supporting the notion that OAPS represents a distinct subset within APS [24].

Antiphospholipid Antibody and Thrombosis

The deep veins of the lower extremities were affected by venous thrombosis represents the most frequent form of venous involvement and can potentially progress to pulmonary embolism, consequently causing pulmonary hypertension. Other sites susceptible to venous thrombosis encompass the renal, pelvic, hepatic, mesenteric, axillary, portal, sagittal, ocular, and inferior vena cava areas. Arterial thrombosis may encompass arteries of any size, ranging from aorta to small capillaries. Transient ischemic events (TIAs) or ischemic stroke are the most commonly observed arterial manifestations of APS, particularly in young patients lacking other atherosclerosis risk factors, which should prompt consideration for APS. Additional sites prone to arterial thrombosis may include the brachial, retinal, peripheral arteries, mesenteric, and coronary. The presence of arterial thrombosis is indicative of a poor prognostic due to the heightened risk of recurrence in such cases [10].

Platelet activation and the production of procoagulants, such as von Willebrand factor (vWF), are well-known effects of aPL, contributing to the development of thrombosis. Despite this understanding, the precise mechanism behind these processes remains unclear. A study conducted by Levy et al. investigated 88 hospitalised patients with APS, comprising individuals with a history of thrombosis, those without aPL but with a history of thrombosis, and healthy controls [25]. The results revealed that APS patients with a history of thrombosis exhibited heightened platelet adhesion and aggregation compared to both patients without a history of thrombosis and healthy group, both of whom displayed normal outcomes (P<0.01). Further analysis showed that preincubation with recombinant vWF fragment RG12986 inhibited platelet adhesion and aggregation in a specific subgroup of APS patients with thrombotic events, as well as in controls. Nevertheless, no correlation was established between the type of aPL and the occurrence of thrombosis in APS patients. The study suggests that platelet activation in APS involves at least two distinct mechanisms. One mechanism is associated with platelet activation induced by aPL. These findings contribute to a better understanding of the complex interplay between aPL, platelet activation, and thrombosis in patients with aPL [25].

In vitro models have predominantly demonstrated the potentially pathogenic mechanisms underlying thrombus formation. However, insights gained from in vivo thrombosis models, which involve the induction of thrombosis through chemical, mechanical, or photochemical trauma in both mice and hamsters, have shed light on the role of aPL in enhancing thrombus formation. These studies have demonstrated that aPL can significantly augment the formation of blood clots in both venous and arterial systems. Moreover, experiments involving the passive infusion of human aPL immunoglobulin G (IgG), combined with a small quantity of lipopolysaccharide (LPS), have led to clotting events observed in the mesenteric microcirculation of rats. Furthermore, within arterial endothelial cells, the infusion of aPL has been shown to induce alterations in the expression levels of endothelial adhesion molecules. Additionally, there is an observed upregulation in the expression of tissue factor (TF) and nitric oxide (NO), further elucidating the intricate mechanisms by which aPL contributes to thrombotic events. These effects were observed both with and without dimeric β2GPI, highlighting the crucial role of aPL in instigating vascular abnormalities. Platelets were implicated in arterial photochemical-induced thrombus formation, while mice monocytes passively injected with aPL displayed heightened TF expression. These findings underscore the diverse ways in which aPL contributes to thrombus formation and vascular abnormalities, providing valuable insights into the intricate interplay of these antibodies in the thrombotic processes [26].

Pathogenic interactions of aPL with human β2GPI and molecules from mice, rats, and hamsters have been confirmed through in vivo models. Thrombotic effects were induced in vivo using affinity-purified β2GPI IgG and were subsequently inhibited by specific absorption of β2GPI activity. Based on these experimental findings, the subpopulation of antibodies responsible for the thrombotic manifestations of APS is identified as anti-β2GPI-dependent aPL. Notably, β2GPI-dependent LA is reported to exhibit a stronger correlation with thrombosis compared to LA in general [26].

The two most thoroughly studied antigens of aPLs are β2GPI and prothrombin. Various proposed physiological functions of β2GPI include apoptotic cell clearance, binding to oxidised low-density lipoproteins, and interaction with coagulation factor XI. Prothrombin, the precursor of thrombin, plays a crucial role in coagulation. Most LAs target either β2GPI or prothrombin. Anti-β2GPI antibodies have been incorporated into the consensus classification criteria for APS. Ongoing research investigates the clinical utility of anti-prothrombin enzyme-linked immunosorbent assays (ELISAs) [3].

In a study conducted by Forastiero et al., the involvement of anti-β2GPI and anti-PT antibodies in the thrombotic risk was examined in a cohort of 194 consecutive patients displaying persistent LA and/or aCL [27]. The findings indicated that patients testing positive for anti-β2GPI exhibited a higher thrombosis rate compared to APS patients without anti-β2GPI (8.0% vs. 3.1% per patient-year). Similarly, individuals with positive anti-PT demonstrated a higher thrombosis rate compared to those without anti-PT (8.6% vs. 3.5% per patient-year). The presence of IgG anti-β2GPI and anti-PT in patients with LA and/or aCL, particularly those with LA, serves as a predictive factor for an elevated risk of thromboembolic events [27].

**Figure 1:**
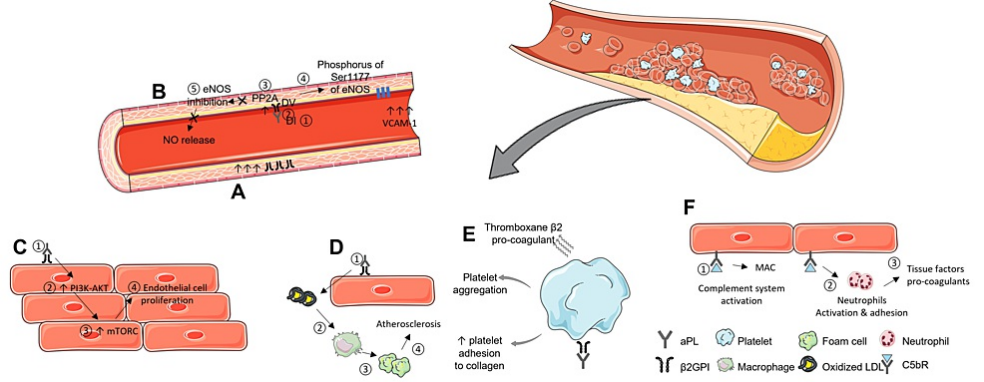
The prothrombotic mechanisms facilitated by aPL A) Second hit: inflammatory insult (infection, trauma, and surgery), B) first hit: eNOS inhibition-mediated endothelial dysfunction, C) intimal hyperplasia vasculopathy, D) accelerated atherosclerosis, E) platelet activation, F) complement activation and neutrophils activation and adhesion. The artwork is modified from [26]. eNOS – endothelial nitric oxide synthase, NO – nitric oxide, PP2A – protein phosphatase 2A, DV – large lysine loop domain, DI – N-terminal domain, VCAM-1 – vascular cell adhesion molecule 1, PI3K-AKT – phosphoinositide-3-kinase-protein kinas AKT, mTORC – mammalian target of rapamycin complex, MAC – membrane attack complex of complement, aPL – antiphospholipid antibodies, β2GPI - β2-glycoprotein I antibodies, LDL – low-density lipoprotein

The procoagulant mechanisms initiated by aPL stem from their ability to interact with phospholipid-binding proteins located on the cell membranes of various cell types. According to the hypothesis, when the antibody binds to its corresponding antigen, it forms a complex that interferes with the integrity of cell membranes. This disruption subsequently triggers signalling pathways that extend to the cell nucleus (Figure 1). Perturbed cells, depending on their biological functions, might elicit different responses, thereby contributing to the diverse clinical manifestations observed in APS [26].

Antiphospholipid Antibodies and Recurrent Miscarriages

A haemostatic abnormality was discovered in 94% of cases with recurrent miscarriages. Among these, approximately 67% were diagnosed with APS, comprising 81.2% with aCL and 4% with LA, respectively. In approximately 55% of patients experiencing recurrent miscarriage, a procoagulant defect was identified as a contributory factor to placental vascular occlusion [28].

In primary APS singleton pregnancies, aCL antibodies were the predominant aPL identified. Nevertheless, the existence of anti-β2GPI was linked to the lowest rate of live births and the highest occurrence of conditions like preeclampsia, intrauterine growth restriction, and stillbirth, in comparison to the presence of aCL antibodies or LA alone [29].

Reflecting the thrombophilic nature of aPL, the initial hypothesis suggested that intraplacental thrombosis, causing impaired maternal-fetal blood exchange, constituted the primary pathogenic mechanism leading to fetal loss. Reports have documented instances of placental thrombosis and infarction, indicating potential complications associated with APS during pregnancy. Furthermore, in vitro investigations have provided insights into how aPL may contribute to the development of a procoagulant environment within the placenta. These encompass the capacity of aPL antibodies to disrupt the anticoagulant annexin A5 shield on trophoblast and endothelial cell monolayers. In alignment with these in vitro observations, the distribution of annexin A5 covering the intervillous surfaces was notably less extensive in the placentas of women positive for aPL compared to those lacking these autoantibodies [26].

Pregnant naïve mice subjected to repeated intraperitoneal injections of substantial quantities of human IgG with aPL activity exhibited pronounced inflammatory damage in the placenta, resulting in fetal resorption and growth retardation. This damage was characterized by the deposition of human IgG and mouse complement, infiltration of neutrophils, and localized secretion of tumor necrosis factor (TNF). The evidence suggested that complement involvement played a role in aPL-mediated fetal loss in this mouse model, as indicated by the protective effects conferred by the deficiency in specific complement components or in vivo inhibition of complement. In this model, the cleavage product C5a of complement component C5 was identified as the key effector, acting through the upregulated expression of tissue factor on infiltrating placental neutrophils. Notably, the protective effect of heparin in the mouse model was associated with its anticomplement activity rather than its anticoagulant properties [26].

The complement and coagulation pathways display intricate interconnections, and emerging data propose that individuals with antiphospholipid antibodies (aPL) may experience complement activation, acting as a cofactor in the pathogenesis of aPL-associated clinical events. During complement activation, C5a is generated, triggering neutrophil tissue factor-dependent procoagulant activity. In vitro studies indicate that β2GPI, the primary antigen for pathogenic aPL, possesses complement regulatory effects. Furthermore, evidence of aPL-induced fetal loss was observed in wild-type mice but not in mice lacking specific complement components (C3, C5). In the same vein, aPL induced thrombosis in wild-type mice, a response that was diminished in mice deficient in C3 or C6 or in the presence of a C5 inhibitor. Despite elevated levels of complement activation products in the sera of aPL patients, their association with clinical events remains unclear.

**Figure 2:**
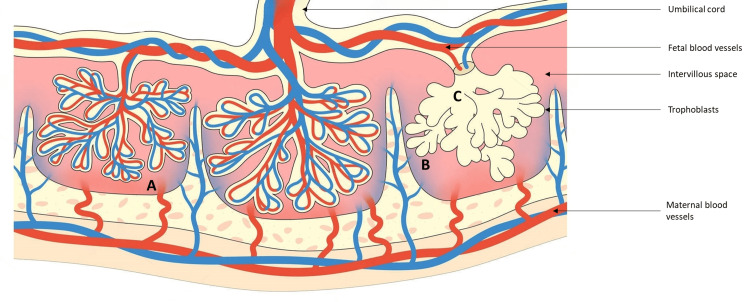
The primary effects of aPL on the placenta A) aPL may initiate placental thrombosis, involving activation of platelet, monocyte, and endothelial cells, along with abnormal disruption of the annexin A5 shield; B) anti-β2PGI antibodies react with trophoblasts, leading to the inhibition of proliferation, differentiation, and induction of apoptosis); C) anti-β2PGI antibodies also react with decidual cells, inducing of a proinflammatory phenotype. The artwork is modified from [26]. aPL – antiphospholipid antibodies, anti-β2PGI – anti-β2-glycoprotein I antibodies.

**Figure 3:**
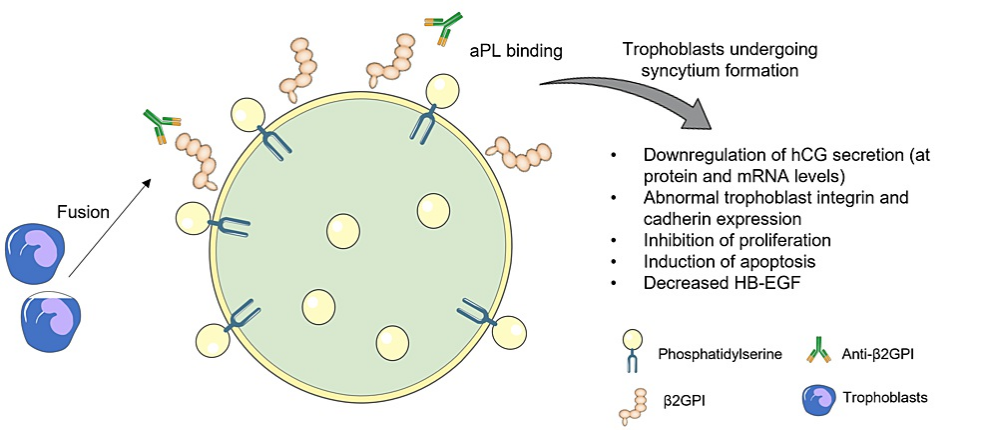
aPL effects on the trophoblasts The artwork is modified from [26]. aPL – antiphospholipid antibodies, hCG – human chorionic gonadotrophin, mRNA – messenger ribonucleic acid, HB-EGF – heparin-binding EGF-like growth factor, β2PGI – β2-glycoprotein I antibodies.

In addition to thrombosis, there exists compelling evidence suggesting that aPL may disrupt normal placentation through alternative mechanisms. These mechanisms involve the direct targeting of both maternal decidua and the invading trophoblast. This suggests that aPL may exert their detrimental effects on pregnancy outcomes not only through their well-documented prothrombotic properties but also through direct interference with the intricate processes involved in the establishment and maintenance of the placenta. Such disruptions in placentation could have profound implications for the overall health and viability of the developing foetus, contributing to the complexity of APS in the context of pregnancy (Figure 2). On the fetal side, aPL, specifically β2GPI-dependent antibodies, bind to human trophoblasts, impacting various cellular functions in vitro (Figure 3). These effects result in cell injury and apoptosis, inhibition of proliferation and syncytia formation, reduced production of human chorionic gonadotrophin, impaired secretion of growth factors, and compromised invasiveness. All these aPL-mediated effects might effects potentially contribute to defective placentation [26].

There is evidence suggesting that aPL can elicit abnormalities in the maternal aspect of the placenta. More precisely, observations drawn from endometrial biopsies have unveiled notable anomalies, including compromised endometrial differentiation and a reduction in the expression levels of complement decay-accelerating factor, recognised as CD55. These pre-conception alterations have the potential to compromise implantation and may predispose individuals to pregnancy failure mediated by complement-related mechanisms. Moreover, β2GPI-dependent aPL has demonstrated the capacity to interact with human stromal decidual cells in vitro, eliciting a proinflammatory phenotype [26]. These findings suggest that complications during pregnancy linked to APS may arise from a spectrum of pathogenic events. Importantly, these events may not solely be attributed to the procoagulant or pro-inflammatory effects typically associated with aPL. This implies that the impact of APS on pregnancy outcomes is multifaceted, involving mechanisms beyond those traditionally considered. Understanding these diverse pathogenic pathways is crucial for comprehensively addressing the complexities of APS in the context of pregnancy.

Diagnosis of APS

The classification criteria for APS were initially formulated in 1999 and dubbed the 'Sapporo criteria.' Subsequently, these criteria underwent revision during the Sydney International Antiphospholipid Antibodies Congress in 2006, becoming known as the updated Sapporo or Sydney criteria. It's important to recognise that these criteria were primarily designed as guidelines for classification rather than diagnostic tools [30,31]. Their primary aim was to establish a standardized framework for identifying individuals likely to have APS. This standardisation enhances the accuracy and consistency of diagnosing APS, thereby facilitating the inclusion of eligible patients in prospective clinical trials aimed at investigating APS management and treatment strategies. Patients currently diagnosed with APS might not necessarily fulfill these classification requirements [32].

Consensus standards for the diagnosis of APS were adopted with the aim of fostering consistency and standardisation across research efforts. These standards are encapsulated in the "Revised Classification Criteria for APS" [11].

Clinical Criteria

The clinical criteria in the diagnosis of APS can be divided into vascular thrombosis and pregnancy morbidity. Vascular thrombosis: This criterion encompasses the occurrence of arterial, venous, or small vessel thrombosis affecting any tissue or organ in the body. It is crucial to note that the diagnosis of thrombosis should be confirmed using objective and validated criteria to ensure accuracy and reliability. Pregnancy morbidity: This category includes various complications related to pregnancy: (a) Unexplained fetal demise: Refers to one or more instances of spontaneous death of a fetus with normal anatomical features, occurring after the 10th week of gestation. The confirmation of normal fetal morphology can be established through ultrasound examination or direct fetal inspection. (b) Pre-term birth: Involves the delivery of a morphologically normal infant before the completion of the 34th week of gestation. This condition arises due to either severe pre-eclampsia or eclampsia, or it displays identifiable signs of placental insufficiency, or (c) Recurrent spontaneous miscarriages are described as the repeated experience of three or more unexplained consecutive spontaneous miscarriages before reaching the 10th week of gestation. To meet this criterion, it is crucial to thoroughly investigate and rule out potential maternal anatomical or hormonal abnormalities, as well as paternal and maternal chromosomal causes, in order to accurately ascertain the underlying causes of these miscarriages [11].

Patient selection for LA Testing

Symptomatic: The assessment for LA should be reserved for patients exhibiting a considerable probability of having APS or presenting with unexplained prolonged activated partial thromboplastin time (aPTT) during routine laboratory evaluation. Guidance for the appropriateness of LA testing is detailed in Table 1 below.

**Table 1:** Appropriateness for LA testing LA – lupus anticoagulants, VTE – venous thromboembolism, ATE – arterial thromboembolism, aPTT – activated partial thromboplastin.

Type of probability	Characteristic
High	Unexplained VTE or ATE in young patients (<50 years old) involving thrombosis at an unusual site. Late pregnancy loss. Any occurrences of thrombosis or pregnancy-related complications in patients with autoimmune disorders.
Moderate	Incidental discovery of prolonged aPTT in an asymptomatic individual. Repeated occurrences of spontaneous early pregnancy loss. Provoked VTE in young patients.
Low	VTE or ATE in elderly patients.

Asymptomatic: It is strongly advised against conducting generalised searches using blood samples from asymptomatic individuals or patient groups not specifically mentioned in these categories. This caution stems from the risk of obtaining false-positive results, a situation that is relatively common due to the assays' poor specificity. Furthermore, once a patient tests positive for lupus anticoagulant (LA), it is crucial to conduct tests on a second occasion, ensuring a timeframe of more than 12 weeks after the initial test. It is essential to collect samples before or without anticoagulant therapy, as this could potentially interfere with the accuracy of the test [33].

In assays where phospholipid content is minimal, the impact of lupus anticoagulants is observed through the prolongation of recalcification times. Despite the availability of numerous assays, they all operate based on this fundamental principle. Confirmation of the presence of lupus anticoagulant may involve applying exogenous phospholipids to demonstrate the normalization of recalcification times [34].

Laboratory Criteria

To establish the diagnosis of APS, it is necessary to confirm the presence of persistent aPL on two distinct occasions, with a minimum interval of 12 weeks between tests. It is imperative to conduct all three aPL tests, which include LA, aCL, and anti-β2GP1 assays. This comprehensive approach is crucial because the specific profile of aPL significantly affects the risk of thrombosis associated with APS. The presence of triple aPL positivity, defined as simultaneous positivity for LA, immunoglobulin G (IgG), and/or immunoglobulin M (IgM) anti-β2GP1, and IgG and/or IgM aCL, correlates with the highest thrombotic risk. aPL with medium to high titres carry clinical significance, particularly concerning thrombotic events, whereas lower titres of IgG or IgM may still be relevant, especially in the context of pregnancy complications. The role of IgM aCL and anti-β2GP1 remains uncertain. Although a multicentre study found no additional benefit in testing for IgM antibodies in thrombotic APS, the combination of positivity for LA, IgG, and IgM was strongly linked with both thrombosis and pregnancy-related complications. This indicates the potential usefulness of this combined positivity in stratifying the risk associated with APS [35]. The recommended approach for testing for aPL is detailed in Table 2 below.

**Table 2:** Testing for aPL *LA testing is performed in different scenarios based on the types of anticoagulants received, two tests using two different principles. Modified from [32]. aAPL – antiphospholipid antibodies, LA – lupus anticoagulant, aCL – anticardiolipin antibodies, β2GP1 – β2 glycoprotein 1, LMWH – low molecular weight heparin, DOAC – direct oral anticoagulant, UFH – unfractionated heparin, ELISA – enzyme-linked immunosorbent assay, IgG – immunoglobulin G, IgM – immunoglobulin M, GPLU – GP liaison unit, dRVVT – diluted Russell viper venom time, aPTT – activated partial thromboplastin time, PL – phospholipid, SCT – silica clotting time, VKA – vitamin K antagonist, TVT/ECT, Taipan snake venom time/Ecarin clotting time

Type	Characteristics
General principle	Simultaneous testing of all three for aPL (LA, IgG/IgM aCL, and anti-β2GP1) at the same time. Repeat tests after at minimum of 12 weeks to confirm persistent aPL-positivity. If using LMWH, samples for LA testing should be taken just before the next dose of LMWH. If feasible, LA testing should follow a brief interruption of DOAC – at least 48 hours after the last dose and longer in patients with renal impairment. Monitoring DOAC levels is advised. The use of DOAC adsorbents requires further investigated in both LA-positive and -negative patient populations. In patients receiving LMWH or UFH, LA testing may be performed if anti-factor Xa activity falls within the therapeutic range, provided the reagents contain heparin neutralisers.
aCL IgG/IgM testing	The aCL/IgM assessment involves ELISA or chemiluminescence techniques and is considered present in medium to high titers when: IgG: 40 GPLU IgM: 40 MPLU Corresponding to the 99^th^ centile for aCL Local verification of the manufacturer’s reference ranges is recommended.
β2GPI IgG/IgM testing	The assay for β2GPI also employs ELISA or chemiluminescence methodologies. A medium to high titer is considered present when it corresponds to the 99^th^ centile for anti-β2GPI. Local verification of the manufacturer's reference ranges is advised.
LA testing*	No anticoagulant: Test such as dRVVT, aPPT (with PL sensitive reagents), and SCT. LMWH/UFH: dRVVT VKA/DOAC: TVT/ECT, which are less influenced by VKAs and anti-factor Xa DOACs. However, their widespread application is contingent upon independent evidence obtained from standardised kits.
Extended aPL testing	Additional aPL testing may include aCL/anti-β2GP1 and antiphosphatidyserine/prothrombin antibodies, assessing domain-1 and -5 β2GP1.

The identification of lupus anticoagulants in laboratory testing is conducted using different principles. LA prolongs recalcification times in assays with small phospholipid content. However, the confirmation of LA existence can be achieved by applying exogenous phospholipid, resulting in normalised recalcification times [34].

**Figure 4:**
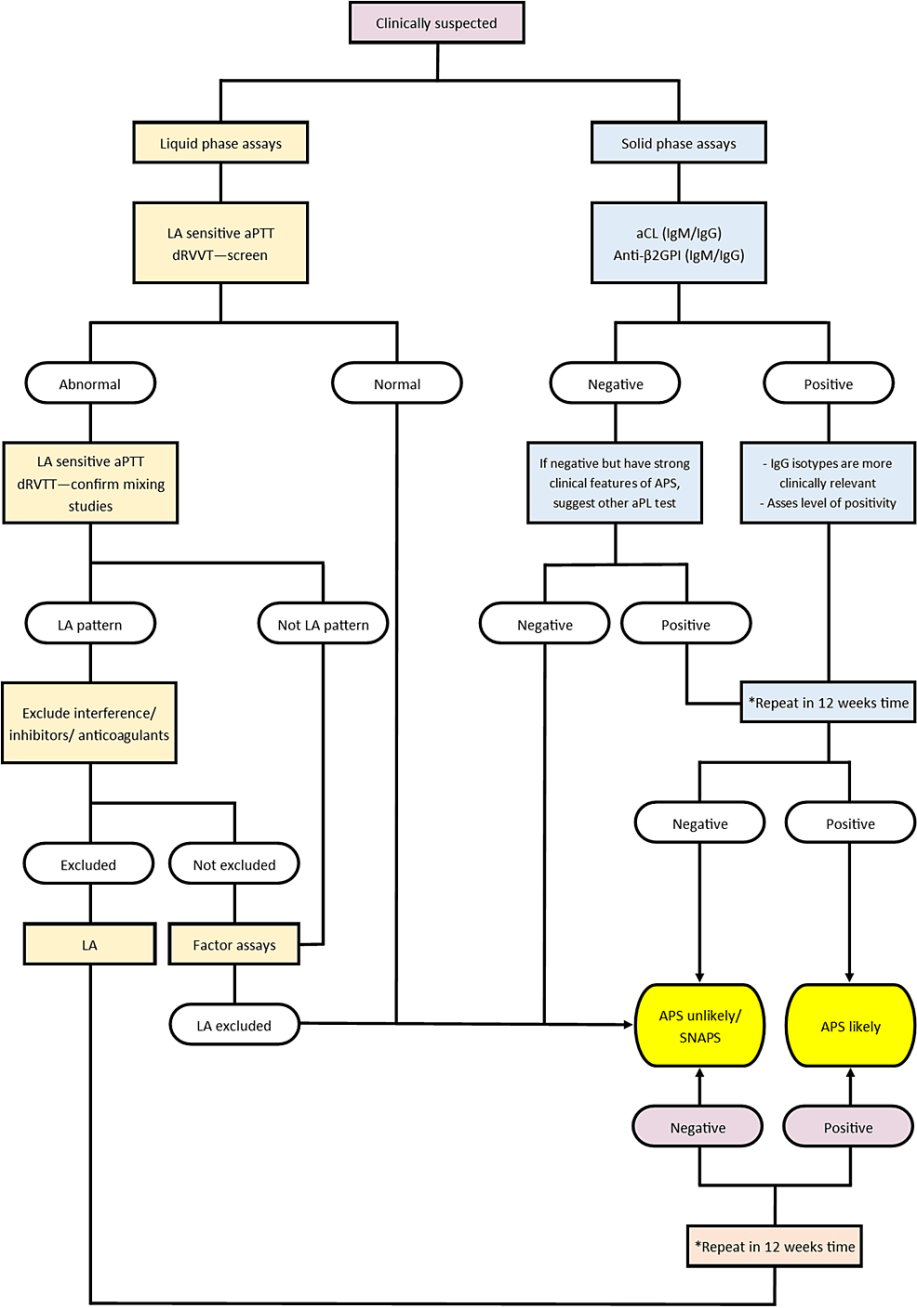
The laboratory testing algorithm for possible APS evaluation *To access persistent and exclude transient or infection-related false-positive; LA – lupus anticoagulant, aPTT – activated partial thromboplastin time, dRVVT – diluted Russell viper venom time, aCL – anticardiolipin antibody, IgM – immunoglobulin M, IgG – immunoglobulin G, β2GPI – beta-2 glycoprotein I antibody, aPL – antiphospholipid antibody, SNAPS – seronegative antiphospholipid syndrome The image is an original artwork created by the authors.

To illustrate the laboratory evaluation for potential APS cases, a suggested algorithm incorporating both solid and liquid phase assays is depicted in Figure 4. Key aspects include the necessity of conducting both solid and liquid phases testing, repetition of tests to confirm persistence or exclude transient results, and the performance of aPL testing after 12 weeks to eliminate falsely negative outcomes at the time of thrombosis [32].

This diagram presents a method that incorporates both solid and liquid phases assays for the assessment of potential APS cases. It emphasizes the need for comprehensive testing procedures, repetition of tests to confirm persistent results, and the necessity of ruling out transient or infection-related false positives. This approach ensures a thorough evaluation for the presence of APS.

aPL profile and thrombotic risk

Diverging from the original Sapporo criteria, the Sydney criteria introduced a novel approach to categorising patients with APS. This reclassification entailed dividing APS patients into two distinct groups: those who tested positive for a single antiphospholipid antibody (such as LA, aCL, or anti-β2GPI) and those exhibiting positivity for more than one laboratory criterion, regardless of the combination. This differentiation stemmed from the acknowledgment that individuals positive for a single antiphospholipid antibody tend to have a lower risk of experiencing thrombotic events or pregnancy complications compared to those with multiple, particularly triple, positivity. Consequently, evaluating patients based on their overall profile of LA, aCL, or anti-β2GPI antibodies, rather than individual test results may provide valuable insights into their thrombotic risk. These observations are consistent with the specific antigenic targets of pathogenic antiphospholipid antibodies, shedding light on the complex interplay between antibody profiles and clinical outcomes in APS.

In 1990, a pivotal subgroup of autoantibodies targeting β2GPI in patients diagnosed with APS was identified. Subsequent investigations illuminated that aCLs linked with thrombotic events predominantly comprise anti-β2GPI autoantibodies exhibiting a robust affinity for cardiolipin. This led to the recognition of two distinct categories of aCL: those dependent on anti-β2GPI activity, associated with thrombotic occurrences and pregnancy complications, and those independent of β2GPI, often encountered in the context of infections. The latter category typically involves transient antibodies and does not carry an inherent risk of thrombosis. Notably, numerous aCL testing kits incorporate a source of β2GPI to enhance sensitivity. Moreover, subsequent studies unveiled that autoantibodies targeting β2GPI can also demonstrate LA activity. Although LA binding to β2GPI and its correlation with thrombosis have been firmly established, the availability of LA assays that detect binding to prothrombin remains limited to research settings. The differentiation between β2GPI-dependent and β2GPI-independent aCLs, as well as LA, bears significant clinical implications. It allows healthcare professionals to distinguish between pathological antibodies associated with thrombotic events and nonpathological antibodies [36].

The mechanism of action differs between anti-β2GPI and anti-prothrombin antibodies in causing LA. Anti-β2GPI antibodies hinder factor V activation by factor Xa through direct interaction. In contrast, anti-prothrombin antibodies compete with factor Xa for phospholipid binding sites. These insights provide clues for understanding the paradoxical association between thrombosis and prolonged clotting time [37].

Laboratory tests focusing on LA, aCLs, and anti-β2GPI antibodies often identify overlapping yet diverse populations of autoantibodies. This diversity may contribute to the varied clinical manifestations observed in APS. LA assays appear to be more effective in detecting pathological aPLs compared to aCLs or anti-β2GPI assays. Moreover, extending beyond the mere identification of whether aPLs bind to β2GPI, it has been observed that different subsets of anti-β2GPI antibodies exhibit recognition of distinct epitopes present on β2GPI molecules. Notably, antibodies binding to β2GPI domain I are associated with thrombotic and pregnancy complications in comparison to those binding to other domains. Laboratory assays detecting domain I-binding autoantibodies often yield triple positivity, identifying higher-risk patients for thrombotic complications. Other predictive variables for thrombosis include persistent aPLs and high titre, which contributed to the inclusion of the Sydney criteria [36].

In addition to binding to β2GPI, aPL may also target other phospholipids and/or protein co-factors. In patients with secondary antiphospholipid syndrome (APS) associated with SLE, these antibodies may be directed against phosphatidylserine, prothrombin (PT), and annexin V. Their presence elevates the risk of thrombotic complications, especially when observed at higher frequencies and concentrations [38].

Autoantibodies recognizing the phosphatidylserine/prothrombin (aPS/PT) complex have been suggested as potential biomarkers for antiphospholipid syndrome (APS). Studies have indicated a robust correlation between aPS/PT antibodies and clinical manifestations, as well as LA. In a study involving 103 patients, persistent positivity for aPS/PT IgG antibodies significantly correlated with APS classification, thrombosis, triple antiphospholipid antibody (aPL) positivity, positive LA results, and a global APS score (GAPSS) >9 points. The persistence of aPS/PT antibodies, aligning with current laboratory classification criteria, is likely to enhance the diagnosis and clinical assessment of patients with APS [39].

The robust correlation observed between LA and IgG/IgM recognizing the phosphatidylserine/prothrombin (aPS/PT) complex suggests that this marker could be valuable in the evaluation of APS. The inclusion of IgG/IgM aPS/PT in diagnostic assessments may improve the overall performance for diagnosing APS, particularly in situations where conventional aPL tests consistently yield negative results. Additionally, IgG aPS/PT might serve as a useful indicator for identifying patients at an increased risk of thrombosis.

Risk of Thrombosis With Positive Testing for One aPL

A systematic review uncovered no discernible connection between aCL and thrombosis, a finding subsequently confirmed by prospective studies. Even when detected at moderate to high titers (aCL >40 IgG or IgM phospholipid units), the presence of aCL alone did not show any association with thrombosis, although it did not demonstrate a link with pregnancy morbidity. Similarly, individuals testing positive solely for anti-β2GPI tend not to display any correlation with thrombosis or pregnancy loss. In contrast, LA appears to exhibit a stronger correlation with thrombosis and pregnancy complications. However, conflicting evidence suggests that isolated LA may not necessarily elevate thrombotic risk [36]. Women with persistent LA face a notably high risk of severe, potentially life-threatening pregnancy complications and adverse outcomes [40].

ELISA assays for aCLs and anti-β2GPI have encountered issues related to poor standardization and unreliable outcomes. In contrast, LA assays demonstrate better standardization and a strong association with the clinical manifestations of APS [36].

Risk of Thrombosis With Positive Testing for Two or More aPLs

Analysis of patient samples collected during the enrollment of the Warfarin in Antiphospholipid Syndrome (WAPS) study revealed that individuals testing positive for LA and β2GPI exhibited an increased risk of overall thrombosis. Various retrospective and prospective investigations have demonstrated that patients displaying positivity in three aPL tests are confronted with increased susceptibility to thrombosis or complications related to pregnancy. In a retrospective analysis involving a cohort of 160 patients with triple-positive aPL tests, the cumulative occurrence of thrombosis escalated to 12.2%, 26.1%, and 44.2% following 1, 5, and 10 years of monitoring, respectively. Furthermore, in a prospective examination comprising 194 patients exhibiting persistent LA and/or aCL, individuals with persistent LA who also tested positive for anti-β2GPI and anti-prothrombin antibodies exhibited the highest incidence of thrombosis, estimated at 8.4% per patient-year. It is imperative to conduct further investigations to ascertain whether the specific profiles of aPL can exert an influence on the classification of antiphospholipid syndrome (APS) and potentially impact clinical decision-making processes. The influence of aPL profiles on thrombotic risk requires additional study.

Thrombotic risk in patients is influenced by additional factors such as the presence of other thrombotic risk factors (such as hereditary thrombophilia, pregnancy, immobilization, and surgery) and the coexistence of SLE, along with the patients' aPL profile. Individuals with SLE are at a heightened risk of thrombosis compared to the general population, and those with isolated but persistently positive aPL tend to face an even greater risk [36].

Management of APS

Asymptomatic Patients

In asymptomatic carriers of aPL, the risk of thrombosis varies depending on the level, titre, type, and quantity of positive aPL antibodies. A prospective study involving aPL-positive patients without prior thrombotic events revealed a comparable thrombosis risk to that of the general population if only one aPL antibody was present (0.7% per year). However, if the patient tested positive for three antibodies, this risk elevated to 5.3% per year. While no evidence supports regular thromboprophylaxis in asymptomatic carriers of aPL, it is recommended to actively manage other modifiable vascular risk factors, promote smoking cessation, and avoid estrogen-containing therapies. In situations involving transient increased thrombotic risk, such as hospitalisation or prolonged immobility, short-term heparin prophylaxis is advised.

For women who are asymptomatic carriers of aPL and have no prior pregnancy-related complications, limited evidence exists. However, several consensus groups recommend close monitoring and the use of low-dose (75 mg) aspirin during pregnancy. In women with coexisting SLE, administering low-dose aspirin also helps reduce the risk of preeclampsia throughout pregnancy [24].

Vascular Thrombosis

For individuals presenting with persistent positive aPLs alongside a history of unprovoked thromboembolism, the administration of lifelong anticoagulation therapy is deemed imperative. Typically, the anticoagulant regimen commences with heparin and progresses to warfarin maintenance. Extensive research has been conducted to ascertain the optimal intensity of anticoagulation. Presently, the established protocol for the long-term management of venous thrombosis in APS necessitates the maintenance of an internationally normalized ratio (INR) within the range of 2-3. This recommendation stems from the findings of two randomized controlled trials, which revealed no substantial advantage in utilizing high-intensity INR (INR > 3) compared to low-intensity (INR 2-3) warfarin in the prevention of recurrent thrombosis. The optimal treatment for arterial thrombosis in APS remains a subject of debate due to the low recurrence rate and the limited number of arterial events observed in these trials.

There is a divergence of opinions among experts regarding the optimal treatment approach. Some propose the combination of warfarin at INR between 2 and 3 along with low-dose aspirin, whereas others argue in favour of warfarin therapy with a higher INR target ranging from 3 to 4. It is crucial to note that as the target dose for anticoagulants increases, so does the risk of bleeding.

Direct oral anticoagulants (DOACs) have been created as substitutes for vitamin K antagonists (VKAs) across different indications. The Rivaroxaban in Antiphospholipid Syndrome (RAPS) study showed that rivaroxaban, a factor Xa inhibitor, is a secure alternative to warfarin for secondary prevention in venous thrombotic APS. Nonetheless, at present, there is no supporting evidence for the utilization of DOACs in managing arterial thrombosis in APS.

In cases of persistent thrombosis while on warfarin, several options can be considered. These options encompass adjusting the INR target to a range of 3-4 in the event of recurrence while maintaining it between 2 and 3. Other strategies may involve incorporating low-dose aspirin (or clopidogrel) into the regimen or transitioning to low-molecular-weight heparin (LMWH). Additionally, supplementary agents that could be contemplated include hydroxychloroquine, which has exhibited both anti-inflammatory and anti-thrombotic properties in cases of SLE; statins, renowned for their anti-inflammatory effects in small cohorts of patients with APS and their ability to reduce the incidence of venous thromboembolism in larger population studies; and sirolimus, an mTOR inhibitor that has demonstrated efficacy in mitigating renal vasculopathy following APS-associated nephropathy. Several case reports and series have suggested potential benefits from rituximab in refractory APS cases. In an open-label Phase IIa descriptive pilot study (RITAPS), rituximab demonstrated success in managing certain non-criteria manifestations (refer to Table 2).

Pregnancy

Pregnant patients with a history of OAPS and no prior thrombosis are advised to consider low-dose aspirin and LMWH. The level of evidence supporting this treatment approach varies concerning different obstetric symptoms associated with aPL. While some studies support aspirin as a standalone therapy, cumulative systematic reviews and consensus papers advocate for a combination of LMWH and aspirin. There are limited evidence-based alternatives to aspirin, and heparin fails to ensure a healthy full-term pregnancy. In cases of SLE/APS, simultaneous medication should be considered to manage disease activity in alignment with pregnancy, including corticosteroids and hydroxychloroquine.

Warfarin is not prescribed for patients with thrombotic APS due to its potential teratogenic effects on the fetus during pregnancy. Upon confirmation of pregnancy, patients should transition to therapeutic heparin. Pre-pregnancy counselling is recommended to inform patients about the potential risks of pregnancy and the necessary therapeutic interventions.

Catastrophic APS

This rare condition is managed based on collective knowledge gathered from the International Catastrophic APS (CAPS) registry. This suggested approach includes intravenous immunoglobulin (IVIG), anti-coagulation, plasma exchange, high-dose intravenous glucocorticoids, and cyclophosphamide, particularly in APS associated with autoimmune rheumatic diseases. In cases where patients do not respond to standard therapy, rituximab might be considered. Additionally, case studies have shown the potential benefits of complement inhibitor eculizumab.

In the management of APS, the approach for APS-positive patients and their treatment regimen is as follows [24]: (a) For individuals with a history of previous VTE but not receiving anticoagulation therapy, options include warfarin with a target INR of 2-3 or DOACs. (b) If there has been a previous VTE episode while on anticoagulation, the recommended course of action is warfarin therapy with a target INR of 3-4. (c) For individuals with a history of previous ATE and no anticoagulation therapy, there exists conflicting expert opinion. However, one approach is to administer warfarin with a target INR of 2-3 alongside low-dose aspirin. (d) In cases of recurrent arterial thromboembolism while on anticoagulation therapy, the preferred treatment is warfarin with a target INR of 3-4. (e) For individuals experiencing recurrent thrombotic events, a combination of low-dose aspirin or clopidogrel along with warfarin may be recommended.

Regarding pregnancy management in aPL-positive women and associated recommendations [24]: (a) For individuals with no history of thrombosis but testing positive for aPL, a cautious approach involving regular monitoring along with the administration of low-dose aspirin is recommended. (b) In cases of SLE combined with APS, treatment typically involves low-dose aspirin alongside LMWH therapy, with the potential addition of hydroxychloroquine. (c) Individuals with a history of previous thrombosis are often prescribed a regimen consisting of low-dose aspirin along with a therapeutic dose of LMWH. (d) For individuals experiencing recurrent early miscarriages, a treatment regimen comprising low-dose aspirin alongside a prophylactic dose of LMWH is commonly recommended. (e) In instances of late fetal loss, severe pre-eclampsia, or previous intrauterine growth restriction (IUGR), a therapeutic approach may involve the administration of low-dose aspirin alongside LMWH therapy.

The potential adjunctive therapies [24]: (a) Statins have shown promise in managing recurrent thromboembolism even in cases where anticoagulation therapy is ongoing, suggesting their potential utility as an adjunctive treatment. (b) Eculizumab, functioning as a C5 inhibitor, has garnered attention for its reported effectiveness in preventing APS-associated thrombotic microangiopathy following renal transplantation and in managing recurrent CAPS, as indicated by case reports. (c) Sirolimus operates by inhibiting B and T cell activation through mTOR inhibition. In renal transplant recipients, the administration of sirolimus has been associated with the absence of APS nephropathy recurrence and a reduction in vascular proliferation. (d) Autologous stem cell transplantation, while showing early promise in the treatment of SLE and APS, is accompanied by rates of adverse events that warrant consideration, based on initial studies.

Prognosis of APS

The results of the 10-year follow-up from the Euro-phospholipid project unveiled a re-thrombosis rate of 15.3% in patients receiving conventional management for APS. While DVT stood out as the most frequent initial thrombotic event, occurrences of arterial thrombotic events increased throughout the disease course. Early pregnancy loss emerged as the predominant obstetric complication, affecting 16.5% of patients. Although 72.9% of pregnancies concluded with the delivery of one or more live infants, a notable degree of fetal morbidity endured, with 48.2% of newborns being born prematurely. Over a 10-year period, a total of 9.3% of patients passed away, with the majority of deaths attributed to severe thrombotic incidents (36.5% myocardial infarction, strokes, and PE, and 10.7% from haemorrhages) [24].

To prevent or reduce the adverse outcomes of APS, it is essential to implement targeted strategies such as early diagnosis, aggressive anticoagulation therapy, and regular monitoring of patients. Prophylactic measures during high-risk periods, such as pregnancy, and patient education about adherence to treatment protocols are also crucial.

The future of APS research aims to enhance understanding of the disease's pathophysiology and improve patient outcomes. Current knowledge includes the identification of aPL and their role in thrombosis, but further research is needed to elucidate the mechanisms behind aPL persistence and variability in clinical manifestations. To achieve this, collaborative international research efforts, longitudinal studies, and advanced genomic and proteomic analyses are necessary. These approaches will help develop more effective treatments and personalized management strategies for APS patients.

## Conclusions

APS represents a prothrombotic condition characterised by diverse symptoms, primarily involving venous and ATE, alongside recurrent pregnancy loss. The array of clinical presentations and the variability in the antibodies crucial for APS diagnosis have posed challenges in studying this condition and have sparked debates regarding the most effective treatments. Individuals with consistently positive aPL, notably those exhibiting triple positivity, face an increased risk of thrombosis and those encountering recurrent thrombotic events despite antithrombotic therapy are also at elevated risk.
